# ENDORSEMENT OF DRIVING AVOIDANCE BEHAVIORS AND COGNITIVE, PHYSICAL, AND VISUAL PREDICTORS AMONG OLDER DRIVERS

**DOI:** 10.1093/geroni/igad104.1928

**Published:** 2023-12-21

**Authors:** Alan Mintz, Lesley Ross, Dustin Souders, Despina Stavrinos

**Affiliations:** Clemson University, Clemson, South Carolina, United States; Clemson University, Clemson, South Carolina, United States; Clemson University, Clemson, South Carolina, United States; University of Alabama at Birmingham, Birmingham, Alabama, United States

## Abstract

Due to age-related changes in visual, physical, and cognitive abilities, certain driving conditions may become more challenging for older adults. Secondary analyses were conducted to assess driving avoidance habits of 72 healthy drivers (65 – 85 years old). Additionally, the physical function (Turn360, Timed Get Up and Go), cognitive (Useful Field of View, Mazes, Trails B, and Wechsler Memory Scale), and visual (visual acuity, Pelli-Robson contrast sensitivity) performance predictors of these habits were explored using linear regression. 59.7% of participants reported avoiding driving in bad weather, 44.4% of participants reported avoiding driving at night, and 40.3% of participants reported avoiding driving during rush hour, making these the most frequently avoided situations. Only 5.6% of participants reported avoiding driving alone and 15.3% of participants reported avoiding driving on high-speed interstates or expressways, making these the least often avoided situations. Women (β = -.393, p = .001) and poorer physical function (β= -.301, p = .018) were associated with greater driving avoidance. Cognition and vision performance did not predict the driving situations. While these results are limited by the high SES of the sample, the results indicate that driving avoidance interventions should consider gender and physical function. Implications and future directions are discussed.

